# Evaluating Sustainable Practices for Managing Residue Derived from Wheat Straw

**DOI:** 10.3390/bioengineering11060554

**Published:** 2024-05-30

**Authors:** Harikishore Shanmugam, Vijaya Raghavan, Rajinikanth Rajagopal, Bernard Goyette, Linxiang Lyu, Siyuan Zhou, Chunjiang An

**Affiliations:** 1 Department of Bioresource Engineering, McGill University, 21111 Lakeshore Road, Sainte-Anne-de-Bellevue, QC H9X 3V9, Canada; harikish97@gmail.com; 2 Sherbrooke Research and Development Center, Agriculture and Agri-Food Canada, 2000 College Street, Sherbrooke, QC J1M 1Z3, Canada; rajinikanth.rajagopal@agr.gc.ca (R.R.); bernard.goyette@agr.gc.ca (B.G.); 3 Department of Building, Civil and Environmental Engineering, Concordia University, Montreal, QC H3G 1M8, Canadazhousiyuan95@outlook.com (S.Z.); chunjiang.an@concordia.ca (C.A.)

**Keywords:** biomass, wheat straw, life cycle assessment, supply chain, greenhouse emissions

## Abstract

Farm leftovers, particularly crop residues, are a key source of renewable energy in Canada. The nation’s robust agricultural industry provides ample biomass, derived from forestry and agriculture resources, for energy generation. Crop residues, such as straws and husks, play a crucial role in this biomass reservoir, contributing to biofuel production and greenhouse gas mitigation efforts. Focusing on supply chains, waste management, and emission reduction, this study evaluates the sustainability of wheat straw, an agricultural biomass by-product. The environmental issues of various approaches to managing agricultural biomass were explored. Following an evaluation of biomass features, conversion methods, and economic and environmental advantages, the results show anaerobic digestion to be the most sustainable approach. Four metrics were examined in relation to social elements, and numerous aspects were considered as inputs in the evaluation of transportation costs. The use of electric trucks versus fuel-based trucks resulted in an 18% reduction in total operating costs and a 58% reduction in consumption costs. This study examined CO_2_ emissions over four different transportation distances. The data indicate that a significant reduction of 36% in kg CO_2_ equivalent emissions occurred when the distance was lowered from 100 km to 25 km. These findings offer insights for creating practical plans that should increase the sustainability of agricultural biomass leftovers.

## 1. Introduction

Agriculture is the main source of the world’s food supply, but it produces a sizable amount of secondary output in the form of agricultural byproducts. Agriculture constitutes one of the most extensive biological sectors and produces abundant biomass, which is a key resource for the bioeconomy [1]. Agricultural byproducts primarily encompass materials released or remaining in various facets of agricultural activities, including production, livestock breeding, and agricultural product processing. These residues contain essential nutrients such as nitrogen (N), phosphorus (P), organic matter, and trace elements crucial for plant growth [2]. The improper disposal of agricultural byproducts can result in significant resource loss and various types of environmental and ecological damage; such disposal also poses substantial challenges to agricultural environmental protection efforts [3]. Proper management and disposal practices are essential to mitigating these adverse impacts.

Agricultural byproducts comprise unused plant components and crop remains, all of which pose challenges to environmental sustainability [4]. Efficient management is essential, with contemporary methodologies emphasizing the utilization of agricultural biomass for energy generation, presenting significant potential for electricity production. Overcoming these obstacles necessitates the development of comprehensive strategies and international collaboration aimed at enhancing resource efficiency and mitigating environmental repercussions on a worldwide level. Agricultural byproducts, generated throughout various stages of production, including pre-harvesting, harvesting, and processing activities [5], have notable potential for biodegradation due to their substantial organic matter content and the presence of diverse macro- and micro-nutrients conducive to microbial growth. These agricultural residues provide promising alternative sources of renewable energy. Through various conversion processes, they may be transformed into valuable products such as biofuels, biogas, bioelectricity, bio-bricks, fertilizer, and biochar [6]. Such multiple usages underscore the dual benefits of addressing waste management concerns while harnessing the potential of agricultural byproducts for sustainable and multifaceted energy production.

The global community confronts two interconnected challenges: the exponential generation of substantial waste and the depletion of fossil fuel reservoirs. Simultaneously, managing agricultural byproducts presents a formidable task, with an estimated annual output reaching 1000 million tons. This double problem highlights the urgency of creating sustainable solutions that will help ease the pressure on the limited supply of fossil fuels while also addressing the growing waste problem [7]. Developing more resilient and sustainable global environments and overcoming these obstacles both require the implementation of efficient agri-waste management systems. If developing countries continue to improve their farming methods, the amount of agricultural byproducts produced worldwide is expected to increase dramatically [8]. The agriculture industry plays a major role in Canada, accounting for around 8% of the country’s overall greenhouse gas emissions. Embracing and applying sustainable methods [9] is essential to effectively managing and reducing the agricultural sector’s overall carbon footprint. Incorporating environmentally conscious practices into farming operations can diminish the industry’s carbon footprint, thereby promoting a more sustainable and eco-friendly agricultural system. Canada produces 82.4 million dry tons of agricultural leftovers annually, on average. A previous study’s results show that the production of agricultural residue is dominated by wheat straw [10].

Wheat straw is composed of a polymeric composite, comprising cell walls consisting primarily of cellulose, which is linear and crystalline in structure, alongside hemicellulose, a branched non-cellulosic heteropolysaccharide, and lignin, which is branched and non-crystalline in nature [11]. Wheat straw stands as a significant and plentiful biomass resource globally. Nevertheless, a large portion of this resource remains unused and is often discarded as waste, leading to substantial air pollution when farmers resort to burning it after the kernel harvest [12]. Many farmers opt to burn abandoned straw directly due to the numerous issues it poses, including mosquito breeding and interference with sowing processes [13]. With the development of the regional economy, advancements in farmland cultivation, and ongoing enhancements in transportation efficiency, an increasing amount of straw is either discarded or incinerated directly [14].

In Canada, the removal of average available crop residues from the land varies across different types of crops. Wheat accounts for the highest amount, with 28 million dry tons per year, followed by corn, barley, oats, and canola (11 million, 8 million, 5 million, and 4 million dry tons, respectively); soybeans, dry peas, and flaxseed also contribute 3 million, 2 million, and 1 million dry tons (respectively) of residues removed from the land annually.

These figures provide insights into the distribution of crop residues and emphasize the significance of crop type in determining Canada’s agricultural waste landscape. The average available crop residues removed from land in Canada from 2001 to 2010 indicate that wheat has the highest percentage among various crop residues [10]. Wheat is globally significant as a major food [15] and nutritional protein source [16], occupying a significant portion of arable land. Various factors, such as high-yielding cultivars, pesticides, and technological advancements, influence wheat output [17]. Notably, the consumption of wheat correlates with population growth, which emphasizes its importance in numerous countries [18]. Agricultural statistics from Agriculture and Agri-Food Canada (AAFC) and Statistics Canada reveal several differences in wheat production parameters for the years 2022–2023 and 2023–2024 [19]. These variations underscore the dynamic nature of agricultural practices and their impact on production outcomes. Nearly 1 kg of wheat straw is produced for every 1.3 kg of wheat grain that is produced [20]. A comparative analysis of wheat straw output at the provincial and national levels has also highlighted Quebec’s and Canada’s significant contributions to wheat and wheat straw production [21].

Statistical data reveal that wheat straw, as a residue, comprised the highest proportion of average crop residues removed from Canadian land between 2001 and 2010. Such statistics emphasize the importance of conducting a life cycle assessment (LCA) to determine the sustainability of wheat straw. In Quebec, the total annual crop residue production amounts to 5.88 million dry metric tons, with a significant portion available for biofuel production after removal from the land [10]. A sophisticated allocation approach is evident in the use of crop residue for livestock, particularly in animal bedding. Based on calculations, the percentage of wheat straw residue that is used for animal feed is about 20.45%. The utilization of a significant portion of collected waste for cattle bedding underscores the diverse applications of agricultural byproducts. This outcome signifies a practical and sustainable approach to utilizing crop residues, enhancing both cattle welfare and the resource efficiency of agricultural practices. Furthermore, by emphasizing the deliberate allocation of agricultural byproducts for the betterment of cattle, the strategy elucidates the intricate balance among various applications of crop residues within the agricultural landscape. Wheat straw is a significant agricultural crop biomass residual in Canada. Similar to other lignocellulosic biomasses, wheat straw is mostly made up of cellulose, hemicellulose, and lignin, with traces of ash and soluble materials called extractives. The overall chemical composition of wheat straw can vary slightly, depending on the wheat variety, the soil, and the environment [22].

This study aims to evaluate the sustainability of wheat straw by paying particular attention to emissions reductions, supply chains, and waste disposal techniques, which can help identify the most effective methods for managing residue produced from wheat straw. To achieve this objective, various waste management approaches will be examined. This novel approach introduces unique combinations of waste management methodologies and assessments using LCA, with a focus on the Canadian setting, filling a significant gap in this area and providing tailored insights that are crucial for directing policy decisions on waste management.

The goal Is to choose the best sustainable course of action by carefully weighing the economic and environmental implications of each approach. A second objective is to scrutinize the effectiveness and sustainability of supply networks for wheat straw by examining the supply chain, from the generation of biomass residue to its eventual use or disposal.

## 2. Materials and Methods

### 2.1. SWOT Study

A SWOT analysis (Strengths, Weaknesses, Opportunities, and Threats) of agricultural biomass crop residue was conducted, with data extracted from an environmental study and categorized into two groups: external factors (Opportunities and Threats) and internal factors (Strengths and Weaknesses) [23]. This analysis was performed to evaluate the potential and challenges linked to utilizing crop leftovers for purposes such as generating bioenergy, enhancing soil health, and implementing sustainable agricultural practices. Several important factors become clear when analyzing wheat straw’s qualities as a resource. Most importantly, its ability to be renewed means that the material can provide long-term sustainability in a range of applications. Also, wheat straw boasts lower emissions, which can help to achieve various environmental goals.

Alongside these strengths, however, wheat straw also presents certain weaknesses; a chief weakness is its restricted availability, which can limit widespread utilization. Transportation challenges also pose logistical hurdles that must be addressed to maximize its efficiency, while the need for nutrient removal poses operational challenges that require careful consideration. Wheat straw offers encouraging prospects for additional research, despite these drawbacks. Its potential for energy generation is particularly noteworthy since it can provide a sustainable substitute for fossil fuels and aligns with the goals of renewable energy generation. The use of this technology in waste management procedures opens up creative responses to environmental problems. The application of wheat straw can be used to produce carbon credits, offering monetary rewards for its implementation. The use of wheat straw does carry risks, however. Wheat straw production may be negatively affected by competing uses for agricultural land, which could draw away resources. Alterations in land use, such as deforestation to facilitate expanded agricultural activities, have the potential to amplify existing environmental issues. Diligent maneuvering within legal frameworks and policies is imperative to sidestep regulatory barriers that might impede the widespread adoption of solutions based on wheat straw.

In conclusion, several benefits and possible uses for wheat straw exist. Its renewable nature, adaptability, and ability to reduce emissions present exciting potential, especially in the domains of energy production, waste management, and the production of carbon credits. To fully capitalize on its benefits, it is essential to overcome several hurdles, including limited availability, complex transportation considerations, and regulatory constraints. Despite these challenges, the numerous advantages and benefits of wheat straw highlight its crucial role in advancing sustainability.

### 2.2. Exploring Methods to Convert Wheat Straw into Valuable Products

**Figure 1:**
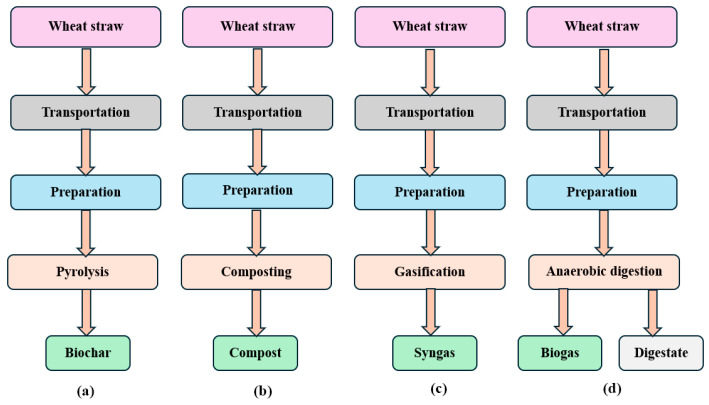
Flowchart of converting wheat straw into useable products by (**a**) pyrolysis, (**b**) composting, (**c**) gasification, (**d**) anaerobic digestion.

Wheat straw, a byproduct of wheat harvesting, can be disposed of using various techniques, depending on the desired outcome and different environmental considerations. The four main techniques for converting wheat straw are anaerobic digestion, pyrolysis, composting, and gasification, which result in biogas, biochar, compost, and syngas, respectively (Figure 1).

The different bioenergy and waste management procedures help to ensure that resources are used sustainably. The wheat straw is initially transported from the harvest center to the facility by a single truck. After transportation to the processing facility, the wheat straw undergoes the preparation phase. This involves essential steps such as harvesting, cleaning, and size reduction. These initial procedures are aimed at readying the straw for further processing. The preparation phase ensures that the wheat straw is appropriately primed for subsequent conversion processes, maximizing efficiency and quality in the production of useful end products like biochar, compost, syngas, and biogas through pyrolysis, composting, gasification, and anaerobic digestion, respectively.

Anaerobic digestion converts organic materials into biogas via microbial activity in an oxygen-free environment. Pyrolysis is the process of heating biomass to generate biochar, which is a carbon-rich substance used in agriculture. Composting, a natural decomposition process that uses oxygen, produces nutrient-rich compost from organic residue. Gasification, a thermochemical process, turns carbonaceous materials into syngas, a flexible gas combination. These approaches not only solve waste management issues but also encourage the long-term generation of renewable energy, resulting in a more ecologically conscious and resource-efficient future.

### 2.3. Life Cycle Assessment

The analytical tool and systematic process known as an LCA is outlined in the ISO 14040–14049 standards [24]. All the fundamental terms, guiding ideas, methodological elements, and real-world LCA applications are covered by these standards. An LCA is used to evaluate the environmental impact and energy efficiency of a system, process, or product from the point of production to the point of disposal [25]. An LCA can offer crucial insights into environmental impacts, notably showcasing significant reductions in greenhouse gas emissions [26,27].

The LCA study comprises four major stages. During the goal and scope definition phase, the study establishes its objectives, concentrating on assessing the sustainability of wheat straw, specifically in waste management and emission reduction, while also defining the boundaries of the assessment. Next, the inventory analysis stage involves collecting comprehensive data on every aspect of wheat straw’s life cycle, from production to use and eventual disposal. During the impact assessment phase, the environmental, social, and economic consequences associated with wheat straw are evaluated in comparison to alternative materials or practices. Ultimately, during the interpretation phase, the gathered data undergo analysis to derive significant conclusions and develop recommendations geared towards improving the sustainability of wheat straw, taking into account all identified impacts. The assessment and condensation of findings within the stages of LCA can provide a platform to determine the basics of the methodology [28]. The examination results in recommendations for possible actions targeted at lessening the product’s environmental impact.

**Figure 2:**
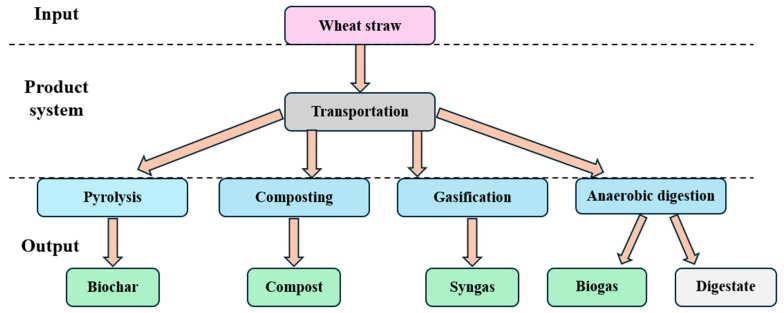
Life cycle assessment of biomass conversion to valuable products.

As noted above, the LCA framework is organized in line with the International Organization for Standardization (ISO) 14040 model, which offers a globally accepted and standardized structure for carrying out LCAs, and the corresponding system boundary is shown in Figure 2. The system boundary classification includes three main phases: input, product system, and output. In the present study, SimaPro 9 software was utilized to conduct the analysis.

### 2.4. Inventory Analysis

A few key factors must be kept in mind while converting wheat straw into biochar, biogas, compost, and syngas through experimentation, including practical viability, safety, and environmental effects. Initially, the designated transportation distance for moving feedstock from the harvest location to the processing facility was set at 100 km in the study. The selected input quantity of wheat straw for biochar, biogas, compost, and syngas production is 1000 kg. The utilization of a straight truck for collecting wheat straw ensures its purity and proper drying to facilitate effective conversion.

Further insights from research on the anaerobic digestion of wheat straw [29] provide additional inputs, including an estimated electricity consumption of 83 kWh and 35 MJ of fuel burned in agricultural equipment, with an additional calculated heat requirement of 394 MJ. A parallel study on pyrolysis conversion [30] provides assumptions of 31 kWh power consumption, 30 MJ fuel consumption by farm equipment, and a calculated heat requirement of 425 MJ. The composting of wheat straw [31] is informed by inputs such as 17.7 kWh of electricity and 35 MJ of fuel burned by farm equipment. Finally, drawn from gasification research [32], the assumptions in the study encompass 83 kWh of electricity for operation, an assumed heat requirement of 650 MJ, and 40 MJ of diesel burned by agricultural machinery. These considerations form the foundation for the subsequent analysis and evaluation of various bioenergy production methods from wheat straw.

Table 1 lists four agricultural processes: anaerobic digestion, pyrolysis, composting, and gasification. The table also includes information about the various processes, including how much wheat straw is used as a raw material, how far it must be transported, how much electricity is used (measured in kWh), and how much fuel is used (measured in MJ). With a power requirement of 83 kWh and 1000 kg of wheat straw, anaerobic digestion offers a balanced solution. The smallest power quantity (31 kWh) but the largest amount of fuel (30 MJ) is required for pyrolysis. Comparatively speaking, composting uses 17.7 kWh of power while burning the same amount of fuel as anaerobic digestion. While gasification and anaerobic digestion are equivalent in terms of power needs for wheat straw, gasification stands out due to its higher fuel consumption of 40 MJ.

### 2.5. Supply Chain Assessment Consideration

An extensive LCA of wheat straw produced data that categorically supported the use of anaerobic digestion as a more sustainable and ecologically friendly option than the alternatives. When compared to alternative utilization techniques, anaerobic digestion demonstrated a considerably lower environmental effect, proving its relative advantage. Research on the social aspect confirms that anaerobic digestion has the least negative effect on the environment. The subsequent endeavor pertains to the refinement of supply chain management concerning anaerobic digestion, with a particular focus on the transportation from the harvest area to the facility.

The transportation method holds significance as it directly impacts the total cost structure. In this thorough investigation of transportation costs, various key parameters are taken into account to offer insight into the overall expenses involved. According to a previous study [33], the average trip distance is assumed to be 100 km, with an average travel speed of 56 km per hour; several assumptions for transportation and operational costs are shown in Table 2.

The process of collecting agricultural biomass residue, such as wheat straw, begins in the fields, following crop harvesting. Specialized equipment (e.g., balers or loaders) is employed to efficiently gather and compress the residue into compact bales or stacks. Once collected, the wheat straw is loaded onto a straight truck for transportation. The straight truck then transports the biomass residue to a processing plant, where it undergoes further processing. Upon arrival at the destination, the biomass residue is unloaded from the truck and is ready to be used in various applications. An essential part of the process is moving biomass from harvesting to facility sites; the expenses involved also affect the system’s sustainability and economic feasibility.

### 2.6. Greenhouse Gas Emission Reduction Strategies by Supply Chain Network Optimization

The optimization of supply chain networks has become a critical strategy in the quickly changing world of modern business, with far-reaching effects that go beyond conventional measures of efficacy and cost. One important consideration in supply chain optimization is environmental sustainability. Below, key areas are discussed where environmental sustainability can be enhanced.

#### 2.6.1. Optimized Transportation by Implementing Green Logistics

“Greenness” has evolved into a code word for a variety of environmental issues [34]. Incorporating ecologically friendly practices into transportation and logistics operations is known as green logistics, and is often referred to as sustainable or eco-friendly logistics. Reducing the environmental effects of supply chain-related transportation operations is the main objective of green logistics. Reducing energy use, carbon emissions, and the ecological footprint overall are all part of this strategy. The usage of automobiles with reduced emissions or vehicles that run on alternative fuels (such as hybrid or electric cars) will be helpful in this regard. By using these alternatives, greenhouse gas emissions and reliance on fossil fuels can be reduced.

#### 2.6.2. Electric Trucks in Supply Chain Optimization

In the subsequent phase of the analysis, the focus is shifted to comparing a conventional fuel-powered straight truck with an electric truck for supply chain purposes. The goal of this comparison is to assess their respective performance and suitability within the broader context of logistics and distribution. In the context of the investigation, the Renault DZE electric truck is reported to be capable of efficiently transporting a maximum load of 16,000 kg [35]. Key assumptions derived from Renault’s specifications include a substantial 300 kWh battery capacity and a long range of 300 km. The energy consumption, computed as 1 kWh/km using the formula (battery capacity/range), sets the foundation for calculating the electric truck’s operational costs. A planned total distance of 500 km and an electricity rate of CAD 0.192 per kWh yield total consumption costs of CAD 96 [36]. This evaluation reflects the consideration given to the electric truck’s technical specifications and energy efficiency, paving the way for a detailed understanding of its operating costs.

## 3. Results

### 3.1. Assessment of Disposal Techniques

To assess and determine the most efficient waste disposal method among anaerobic digestion, gasification, composting, and pyrolysis, an analysis was conducted using SimaPro 9 software. This assessment encompassed the application of three distinct methodologies: the Intergovernmental Panel on Climate Change (IPCC) Global Warming Potential (GWP 100a), the Greenhouse Gas (GHG) Protocol, and Impact 2002+. The primary objective of this analysis was to identify the most appropriate technique for managing agricultural biomass residue.

**Figure 3:**
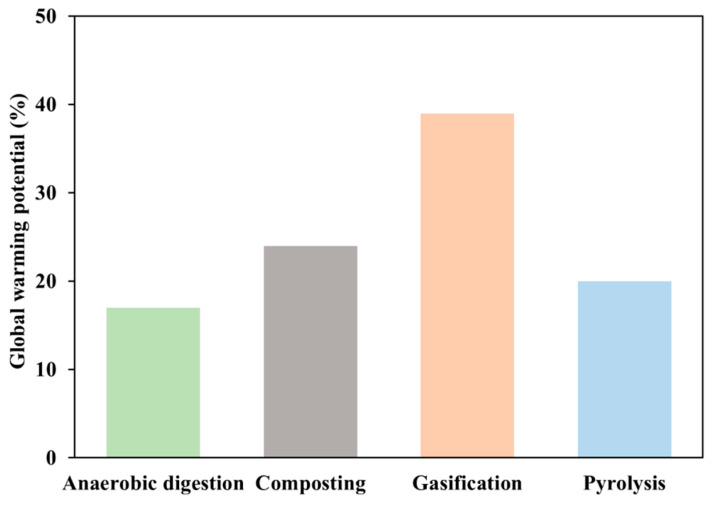
Global warming potential percentage using the IPCC GWP 100a method.

The Intergovernmental Panel on Climate Change (IPCC) Global Warming Potential (GWP 100a), which refers to GWP values calculated over a 100-year period as defined by the IPCC, provides insights into the impacts of various techniques on global warming. According to the findings presented in Figure 3, anaerobic digestion exhibits the least potential for causing global warming, at 17%, followed by pyrolysis (20%) and composting (24%). Conversely, gasification contributes the most to global warming, registering at 39%.

**Figure 4:**
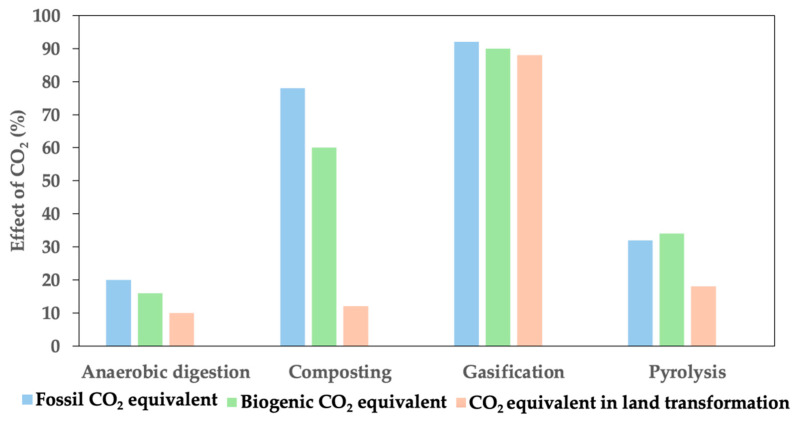
Percentage effect of CO_2_ by Greenhouse Gas Protocol method.

**Figure 5:**
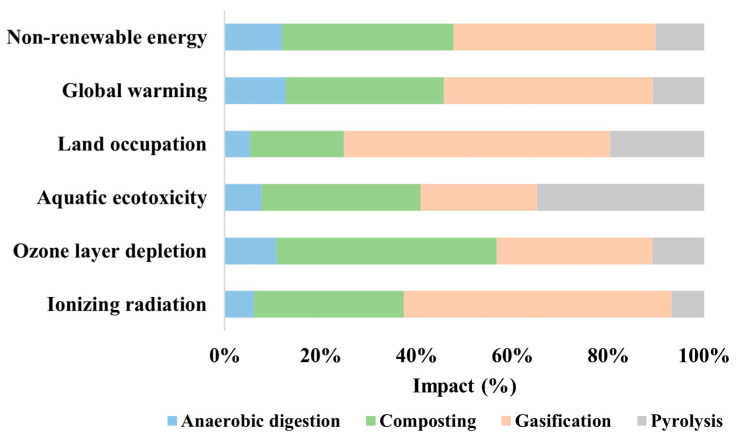
Percentage impacts of six factors using the Impact 2002+ method.

The Greenhouse Gas (GHG) Protocol approach was also integrated into SimaPro, ensuring consistent and accurate emissions calculations in accordance with GHG Protocol criteria. The method describes three key factors: fossil CO_2_ equivalent, biogenic CO_2_ equivalent, and CO_2_ equivalent in land transformation. Figure 4 depicts the outcomes obtained through the GHG Protocol method. The data shown in Figure 5 reveal that anaerobic digestion results in a lower-percentage impact on CO_2_ compared to alternative techniques. Anaerobic digestion shows an average damage percentage of 15% for the three factors, while the pyrolysis technique exhibits an average impact of 28%. Among the three factors, biogenic CO_2_ has the least effect across the four techniques (50%), followed by fossil CO_2_ equivalent (55%).

Impact 2002+ serves as a recognized life cycle impact assessment methodology that is seamlessly integrated into SimaPro 9 software for conducting thorough environmental assessments. This methodology facilitates the evaluation of environmental impacts across diverse categories, including ionizing radiation, ozone layer depletion, aquatic ecotoxicity, land occupation, global warming, and non-renewable energy. Figure 5 provides a visual representation of the outcomes derived from the Impact 2002+ method. Among the six factors, namely ionizing radiation, ozone layer depletion, aquatic ecotoxicity, land occupation, global warming, and non-renewable energy, anaerobic digestion demonstrates the least damage, showing minimal impact on land occupation (8%) and non-renewable energy (26%). The second-best technique is pyrolysis, where ionizing radiation causes the least damage (10%) and aquatic ecotoxicity poses the highest damage (82%). Composting ranks third, with the least damage to land occupation (30%) and the highest damage to ozone layer depletion (85%). Gasification is the least favorable technique, with aquatic ecotoxicity showing the least damage (57%) and non-renewable energy showing the highest (92%). Anaerobic digestion averages about 17% damage, while gasification poses a significantly higher impact at nearly 73%.

After evaluation of the four disposal methods, namely anaerobic digestion, composting, pyrolysis, and gasification, anaerobic digestion emerged as the most promising technique, offering effective waste management with relatively little environmental impact. In contrast, gasification was found to exhibit several drawbacks and showed a higher negative effect on the environment compared to the other methods. These findings underscore the importance of selecting disposal techniques that prioritize environmental sustainability while effectively managing waste.

### 3.2. Social Aspect Analyses for the Agricultural Biomass Waste Disposal Scenarios

**Figure 6:**
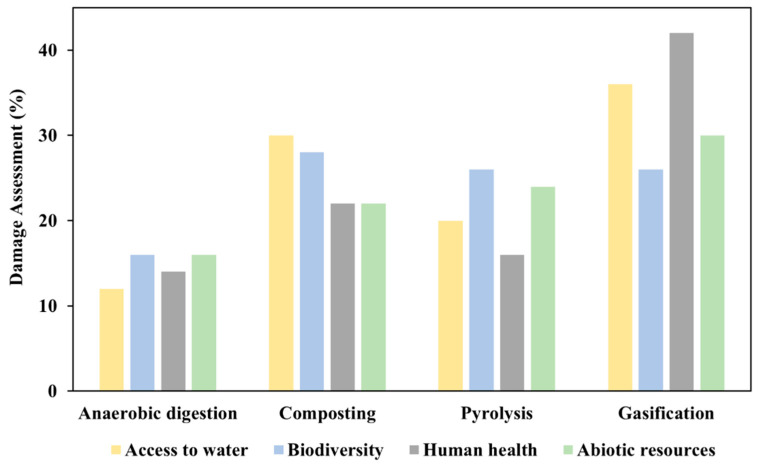
Percentage of damage assessment for the four techniques using EPS 2015dx method.

The disposal of wheat straw is a complex challenge in the context of sustainable farming practices that requires a thorough assessment of its effects on the environment and society. The Environmental Priority Strategies (EPS 2015dx) method is used to evaluate the prevalence and impacts of diseases (notably cancer, asthma, and diarrhea), and it exclusively compares the consumption of abiotic resources across different scenarios related to wheat straw disposal. Figure 6 presents the characterization of damage assessment, encompassing key factors such as biodiversity, human health, abiotic resources, and access to water. This visual representation provides an overview of the assessed damages across these critical dimensions and offers insights into the broader environmental implications associated with the data. The four primary metrics selected for interpretation in the analysis are abiotic resources, human health, biodiversity, and water availability.

The four methods and the appropriate percentages for damage assessment are shown in Table 3. The impact of the four factors on anaerobic digestion appears to be relatively low, with an average of 15%; pyrolysis shows a slightly higher impact, at 22%. When considering the four factors individually, abiotic resources are the least affected, showing a lower impact at 23%. In the following analysis phase, the focus shifted to identifying the most efficient supply chain network for the process, leading to a clear reduction in emissions. This analysis specifically aimed to identify a supply chain setup that would significantly decrease emissions. The goal of this strategic approach is to optimize the process and align it with environmentally friendly practices.

### 3.3. Transportation Analysis Using Electric Truck

**Figure 7:**
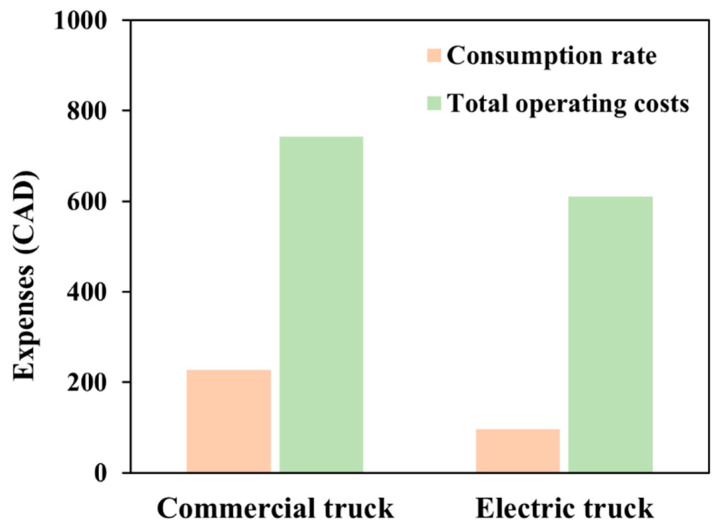
Comparison of consumption rates and total operating costs of electric vehicle and fuel truck.

Upon examining the wider operational context, further cost factors also become relevant. The driver costs for the electric truck are calculated to be CAD 315.7, based on previous fuel truck results. Various other expenses amount to CAD 200. When these elements are considered, the total running costs for the Renault DZE electric vehicle come to CAD 611. Taking into account not only the direct costs of energy consumption but also driver and incidental charges, the current study investigates the financial aspects associated with the integration of electric vehicles in logistics. Both analyses indicate that the overall operating costs of electric trucks are lower compared to those of conventional fuel-powered trucks. Figure 7 shows a comparison of the consumption rate and overall operating costs of an electric vehicle versus a commercial gasoline truck. In terms of consumption rates and overall running expenses, this visual depiction illustrates the performance indicators and enables an evaluation of various features related to electric vs. commercial gasoline vehicles.

Figure 7 clearly shows that the consumption rate and total operating costs are lower for electric vehicles than for commercial fuel trucks. The consumption rate of electric trucks is CAD 96, which is cheaper than that of commercial trucks (CAD 227.5). Similarly, the total operating cost of electric trucks is CAD 611, compared to CAD 743 for commercial trucks. Electric trucks offer a cleaner and more sustainable mode of transportation, benefiting from their inherent efficiency and the potential to utilize renewable energy sources for power.

### 3.4. Optimized Transportation by Reducing the Transportation Distance

**Figure 8:**
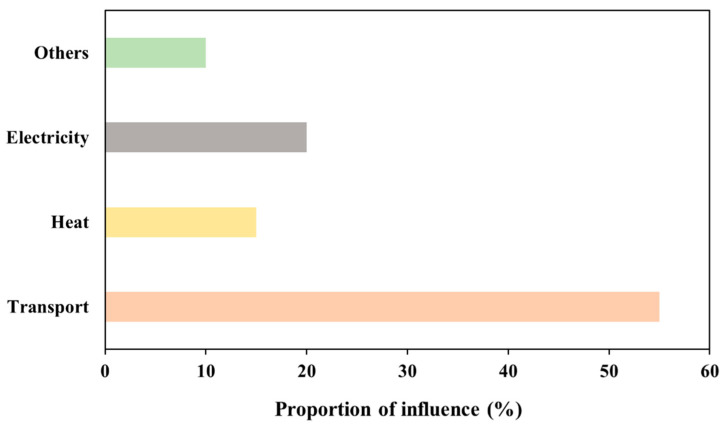
Various factors and the corresponding % kg CO_2_ eq by ReCiPe 2016.

Within the intricate framework of modern supply chain management, minimizing transportation distances has emerged as a pivotal strategy for organizations striving to enhance efficiency and promote environmental sustainability. The ReCiPe 2016 approach stands out among other LCA techniques since it offers the widest range of midpoint effect categories and a worldwide impact calculation mechanism [37]. The proportion of kg CO_2_ equivalent in relation to global warming is the primary target of the approach. (When measuring greenhouse gas emissions, the phrase “kg CO_2_ equivalent” refers to a standard unit that quantifies the potential for global warming of different greenhouse gases in terms of carbon dioxide [CO_2_]). Figure 8 shows the relationship between transport, electricity, heat, and various other factors, along with the percentage of kg CO_2_ equivalent. The analysis was conducted for a transportation distance between the harvest area and the anaerobic digestion plant of 100 km. As shown in Figure 8, nearly 55% of kg CO_2_ equivalent damage to the environment occurs through transport, followed by electricity and heat (20% and 15%, respectively).

**Figure 9:**
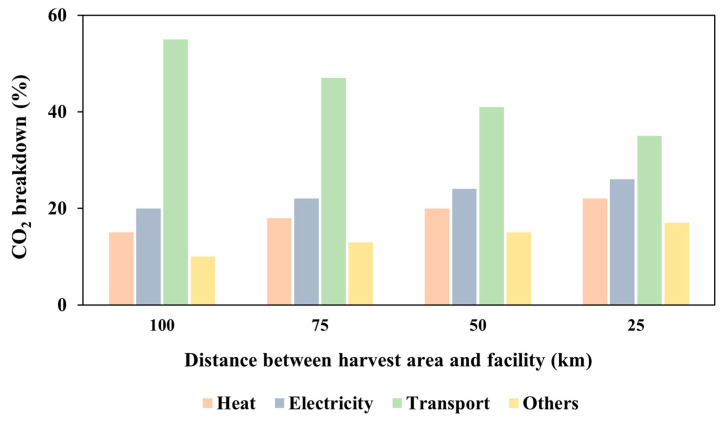
Breakdown of CO_2_ contributions based on four distinct transportation distances.

Figure 9 represents the proportion of kg CO_2_ equivalent of several components at the 100 km transportation distance between the harvest region and the facility. According to the ReCiPe 2016 method, transport accounts for around 55% of the kg CO_2_ generated. The distances of 75 km, 50 km, and 25 km were also assessed between the harvest area and the facility center. According to our findings, transportation distances of 75 km, 50 km, and 25 km were responsible for approximately 47%, 41%, and 35% of the total CO_2_, respectively. The results demonstrate that shorter transportation distances correspond to reduced greenhouse gas emissions. Specifically, when the distance decreases to 25 km, there is a 35% decrease in kg CO_2_ equivalent emissions. Furthermore, shortening the distance from 100 km to 25 km results in an approximate 36% reduction in kg CO_2_ equivalent emissions.

## 4. Conclusions

The main purpose of this study was to evaluate several processing methods for disposing of wheat straw, including gasification, pyrolysis, anaerobic digestion, and composting, to identify the best approach. Anaerobic digestion is the least harmful choice in terms of the environment and has the least effect on global warming when compared to the other processes. The findings, elucidated through the application of an LCA, were followed by an analysis aimed at optimizing the supply chain network to convert wheat straw into biogas through anaerobic digestion. This method was chosen for its significantly lower environmental impact compared to alternative techniques. Anaerobic digestion involves the decomposition of organic matter in the absence of oxygen, yielding biogas as an environmentally friendly energy source. Commencing with the assessment of the conventional supply chain model, a comparison followed on the potential conversion of conventional fuel vehicles into electric trucks. This study presents a groundbreaking perspective by adopting a standardized set of parameters across four waste management methodologies, including a fixed transportation distance of 100 km and a consistent quantity of 1000 kg of wheat straw, using a straight truck for EPS transportation. To ensure precise analysis, the study supplements assumed data with precise values from the literature, enhancing the reliability and relevance of its findings and distinguishing it from previous studies. The results highlight the benefits of adopting electric vehicles over conventional ones in terms of sustainability of the economy and environment. Compared to commercial vehicles, which have a higher rate of CAD 227.5, electric trucks showed a lower consumption rate of CAD 96.5. Electric trucks also have overall operating costs of CAD 611, compared to CAD 743 for commercial trucks. The study also analyzed the resulting kg CO_2_ emissions across various transportation distances, encompassing 25 km, 50 km, 75 km, and 100 km. The findings revealed a significant decrease of approximately 36% in kg CO_2_ equivalent emissions when the distance was reduced from 100 km to 25 km. These results provide valuable insights for developing effective strategies to improve the sustainability of agricultural biomass residues.

## Figures and Tables

**Table 1:** Common factors and associated assumptions for several disposal techniques.

Technique	Wheat Straw (Raw Material) (kg)	Transportation (km)	Power (kWh)	Fuel Burnt (MJ)
Anaerobic digestion	1000	100	83	35
Pyrolysis	1000	100	31	30
Composting	1000	100	17.7	35
Gasification	1000	100	83	40

**Table 2:** Operational costs and assumptions for transportation.

Assumption	Value
Transportation distance	100 km
Type of truck	Straight truck
Harvesting season	Spring and summer months (June, July, August, and September)
Study duration	120 days
Analysis period	5 days
Average loading/unloading time	1.5 h
Total distance covered	500 km
Average daily driving time	1.78 h
Cumulative average driving time	8.9 h
Fuel consumption rate per vehicle	37 L/100 km
Driver base pay	19.25 CAD/h
Fuel cost per liter	CAD 1.50
Fuel consumption cost	CAD 227.5
Miscellaneous costs	CAD 200
Total vehicle operational costs	CAD 743

**Table 3:** Percentage of damage assessment for the four techniques using EPS 2015dx method.

Technique	Access to Water	Biodiversity	Human Health	Abiotic Resources
Anaerobic digestion	13	16	14	16
Pyrolysis	20	26	17	24
Composting	30	28	22	22
Gasification	36	26	42	30

## Data Availability

The data may be available from the authors upon reasonable request.

## List of Abbreviations and Acronyms

CAD	Canadian dollar
CO_2_	Carbon dioxide
EPS	Environmental Priority Strategies
GHG	Greenhouse gas
GWP	Global warming potential
IPCC	Intergovernmental Panel on Climate Change
ISO	International Organization for Standardization
kg	Kilogram
km	Kilometer
kWh	Kilowatt hour
LCA	Life cycle assessment
MJ	Megajoule
N	Nitrogen
P	Phosphorus
SWOT	Strengths Weaknesses Opportunities Threats
